# Comparison of the efficacy and safety of neoadjuvant PD-1 inhibitors plus chemotherapy vs targeted therapy plus chemotherapy in locally advanced hypopharyngeal squamous cell carcinoma

**DOI:** 10.3389/fimmu.2024.1466310

**Published:** 2024-10-31

**Authors:** Wen Gao, Lifei Feng, Xinming Zhao, Zishi Huang, Duoxuan Chen, Gaofei Yin, Wei Guo, Qi Zhong, Xiaohong Chen, Jugao Fang, Yang Zhang, Zhigang Huang

**Affiliations:** Department of Otolaryngology Head and Neck Surgery, Beijing Tongren Hospital, Capital Medical University, Beijing, China

**Keywords:** PD-1 inhibitors, hypopharyngeal squamous cell carcinoma, immunotherapy, comparison of efficacy, drug response

## Abstract

**Objective:**

To investigate the clinical efficacy, preservation of laryngeal function, and safety differences between PD-1 inhibitors combined with chemotherapy, and targeted therapy combined with chemotherapy in LA HPSCC patients.

**Methods:**

This was a retrospective analysis of patients with LA HPSCC treated at Beijing Tongren Hospital, Capital Medical University from October, 2020 to March, 2024. A total of 110 eligible patients were included, 56 in the PD-1 inhibitors combined with chemotherapy group (Group A), and 54 in the targeted therapy combined with chemotherapy group (Group B). Relevant clinical data were collected, and the clinical efficacy, preservation of laryngeal function, complete response (CR) rate, pathological complete response (pCR) rate, major pathological response (MPR), and treatment-related adverse events (TRAEs) of the two groups were analyzed and compared.

**Results:**

In both groups A and B, the objective response rate (ORR) and disease control rate (DCR) were similar with no significant differences, but the pCR rate in Group A was much higher than that in Group B, at 37.5% and 7.4%, respectively (p<0.001). The rate of primary tumor downstaging in group A was much higher than that in group B (76.8% vs. 38.9%) as well (p<0.0001). In addition, the 1y-OS rate in group A was 95.7%, compared to 87.0% in group B (p=0.106, HR=0.34; 95% CI: 0.114-1.013), and the 1y-PFS rate was 89.4% in group A compared to 85.2% in group B (p=0.399, HR=0.675; 95% CI: 0.275-1.659). Furthermore, the larynx function preservation rate was significantly higher in group A at 85.7%, compared to that of group B at only 66.7% (p=0.019). There were no deaths due to TRAEs in either group, and there was no significant difference in the incidence of grade 3-4 TRAEs between the two groups either (p=0.77). The main TRAEs in Group A were metabolism and nutrition disorders (52/56, 92.9%) and, in Group B were blood and lymphatic system disorders (40/54, 74.1%).

**Conclusions:**

PD-1 inhibitors combined with chemotherapy showed better short-term efficacy compared to targeted therapy. Additionally, a trend toward improved long-term survival was observed with PD-1 inhibitors but not with targeted therapy. Results for both groups indicate that neoadjuvant therapy is both safe and manageable.

## Introduction

Head and neck cancer is the seventh most common malignant tumor globally, and hypopharyngeal cancer is one of the more common and highly malignant tumors in the head and neck (3-4% of all head and neck tumors) (1). Among hypopharyngeal cancer patients, 90% suffer from squamous cell carcinoma (2). Due to the lack of specific symptoms and the tendency for hypopharyngeal squamous cell carcinoma (HPSCC) to metastasize to the neck lymph nodes, up to 85% of patients may already have locally advanced disease by the time they first present (3). These patients have a poor overall prognosis even after receiving surgery, radiation therapy, chemotherapy, and targeted therapy, with a recurrence rate of up to 50% and a 5-year overall survival rate below 40% (4). The main treatment options for HPSCC include surgery, radiation therapy, chemotherapy, targeted therapy, and immunotherapy. Most patients with LA HPSCC require a total laryngectomy, which results in permanent loss of laryngeal function and severely affects their quality of life.

Previous phase III studies of neoadjuvant chemotherapy in HPSCC have shown that preoperative neoadjuvant treatment can reduce tumor burden, thereby achieving preoperative downstaging, increasing the chance of preserving organ function during surgery, and improving the rate of laryngeal preservation in HPSCC, but no improvement in overall survival has been observed (5, 6). In clinical practice, suitable neoadjuvant therapy regimens are usually selected based on the specific tumor characteristics of each patient, with the hope of reducing the tumor size to allow for complete resection of the affected tissue while also achieving tumor control and ensuring patient survival, preserving voice function, and improving quality of life. Cetuximab is now approved for first-line treatment of recurrent and/or metastatic head and neck squamous cell carcinoma (R/M HNSCC), and studies show that ORR with cetuximab combined with chemotherapy (EXTREME regimen) can reach 36.3% (7). Previous neoadjuvant studies have only explored data from oral/pharyngeal cancers, and there is currently a lack of data related to hypopharyngeal cancer.

In addition to cetuximab, nimotuzumab is an IgG1 monoclonal antibody that targets the EGF receptor and can block the binding of EGFR to its two main ligands (EGF and TGF-α), inhibit EGFR phosphorylation, terminate signal transduction towards the cytoplasm, and thus inhibit cancer cell proliferation and induce cancer cell apoptosis (8, 9). Its mechanism is similar to that of cetuximab, and it has been approved for the treatment of head and neck cancer in 24 countries around the world. Nimotuzumab thus has further exploratory value in locally advanced hypopharyngeal cancer.

Based on the KEYNOTE-048 study (7), PD-1 inhibitors have become the standard first-line treatment for advanced HNSCC, and there has been extensive exploration of PD-1 inhibitors in LA HNSCC as a new adjuvant treatment as well. The combination of pembrolizumab and chemotherapy has been shown to prolong the duration of pathological response (DOR) and overall survival in patients with R/M HNSCC. However, its efficacy in the initial treatment of LA HNSCC remains to be elucidated (10). A phase II clinical trial (11) reported the efficacy and safety of neoadjuvant chemotherapy combined with PD-1 inhibitors in treating resectable III-IVB stage HNSCC patients. The results showed an ORR of 96.7%, the pCR rate of 37.0% and an MPR rate of 74.1%. The median follow-up time was 16.1 months, and the 1-year disease-free survival (DFS) rate was reported at 95.8%. Additionally, in a phase II clinical trial of neoadjuvant PD-1 inhibitors combined with chemotherapy for resectable LA HNSCC patients (NCT03174275), a total of 35 patients received treatment. The pCR rate and progression-free survival (PFS) rate were 29% and 49%, respectively, with a 1-year DFS rate of 83.8% (12). In 2023, a prospective single-arm, single-center clinical trial (ChiCTR2200055719) demonstrated that 22 patients with LA HNSCC were treated with pembrolizumab in combination with cisplatin and paclitaxel, achieving a pCR rate of 36.4% and a laryngeal function preservation rate of 90.9% (13). Another prospective single-arm, single-center clinical trial reported the results of PD-1 inhibitor combination with albumin-bound paclitaxel, platinum, and fluorouracil for the treatment of LA HNSCC, reporting an ORR of 85.7% and a pCR rate of 42.9% (14). That same year, the results of a phase II clinical trial (ChiCTR2000033506) were reported for toripalimab combined with albumin-bound paclitaxel and cisplatin in the treatment of LA HNSCC patients, with an ORR of 92%, a median follow-up time of 17 months, a 1-year overall survival rate of 96.0%, and a 1-year DFS rate of 88% (15). The preliminary results of these studies have been encouraging, making PD-1 inhibitors a potentially effective treatment option for hypopharyngeal cancer.

In the present study we conducted a retrospective analysis to compare the survival benefit, preservation of laryngeal function, and safety of immunotherapy combined with chemotherapy as neoadjuvant treatment to targeted therapy combined with chemotherapy in patients with locally advanced hypopharyngeal cancer directly.

## Materials and methods

### Enrolled patients

This was a retrospective, single-arm, single-center clinical trial. Patients who had recently treated for LA HPSCC at the Department of Otolaryngology-Head and Neck Surgery, Capital Medical University Affiliated Beijing Tongren Hospital, from October 2020 to March, 2024 and received at least two cycles of neoadjuvant therapy were eligible. The inclusion criteria were as follows: (1) Pathological diagnosis of squamous cell carcinoma of the hypopharynx; (2) Age 18 years or older; (3) No prior treatment; (4) History of immunotherapy combined with chemotherapy or targeted therapy combined with chemotherapy as a new adjuvant treatment scheme at our hospital; (5) Locally advanced hypopharyngeal cancer (stage III-IV according to the AJCC 8^th^ edition staging system); and (6) Both preoperative and postoperative imaging examinations showing measurable target lesions. The exclusion criteria were: (1) Pathological diagnosis of nonsquamous cell carcinoma; (2) History of previous surgery, radiotherapy, chemotherapy, immunotherapy, or targeted therapy; (3) Lack of clinical medical records; and (4) Loss to follow-up.

This study was conducted in accordance with the ethical principles outlined in the Helsinki Declaration. The Institutional Review Board of Beijing Tongren Hospital, Capital Medical University, approved the study protocol (ethical approval number: TRECKY2021-049), and informed consent was obtained from each patient at follow-up after they were informed of the study.

### Methods

Patients were divided into two groups., Group A received pembrolizumab plus TP regimen (paclitaxel liposome + nedaplatin), administered every 3 weeks for 2-3 cycles. The pembrolizumab was administered intravenously at a dose of 200 mg on day 1; Paclitaxel liposome was administered intravenously at a dose of 135-175 mg/m^2^ on day 2; and nedaplatin was administered intravenously at a dose of 80-100 mg/m^2^ on days 3-5. Group B received a nimotuzumab plus TP regimen, administered every 3 weeks for 2-3 cycles (**Table 1**). Post-treatment assessment was conducted according to RECIST 1.1 criteria. Patients achieving a complete response (CR) underwent radiation therapy, and those without a CR underwent surgical treatment followed by standard postoperative therapy. The primary endpoint was pathological complete response (pCR) rate (defined as absence of residual invasive squamous cell carcinoma within the primary tumor specimen on resection), and the secondary endpoints were objective response rate (ORR), disease control rate (DCR), 1-year progression free survival (1y-PFS) rate, 1-year overall survival (1y-OS) rate, CR rate, and the proportion of TRAEs.

**Table 1:** The drug regimens of the two groups.

Treatment Group	Therapeutic regimen	Dose	Time	Course of treatment
Group A	pembrolizumab	200 mg	Day 1	21 days as a cycle, 2-3 cycles.
	paclitaxel liposome	135-175 mg/m2	Day 2	
	nedaplatin	80-100 mg/m2	Day 3-5	
Group B	nimotuzumab	200 mg	Day 1,8,15	21 days as a cycle, 2-3 cycles.
	paclitaxel liposome	135-175 mg/m2	Day 2	
	nedaplatin	80-100 mg/m2	Day 3-5	

### Statistical analysis

SPSS version 26.0 and GraphPad Prism version 8.0.2 were used to perform all statistical analysis. Categorical data were expressed using proportions or rates prior to all calculations, and the chi-squared test was used to compare differences between the two groups of patients. Additionally, the Kaplan-Meier method was used to draw the survival curves for PFS and OS, and the log-rank test was used to analyze intergroup differences. A p-value < 0.05 was considered to indicate a statistically significant test result for all tests.

## Results

### Patient characteristics

Through the inclusion of exclusion criteria, a total of 110 patients with locally advanced hypopharyngeal cancer who received at least 2 cycles of neoadjuvant therapy. Among them, Group A collected 56 patients, Group B collected 54 patients. The relevant clinical characteristics of Group A and Group B were analyzed by chi-squared test, and the results (**Table 2**) show that there were no statistical differences in age, gender, smoking history, drinking history, clinical stage, TN stage, anatomical location, or tumor pathological differentiation (all P>0.05). Furthermore, the majority of patients in both groups are male, and those with a history of smoking and drinking accounted for about 80% of all patients.

**Table 2:** Baseline patient demographic and clinical characteristics [n (%)].

Characteristics	Treatment Group		P value
	A (n=56)	B (n=54)	
Age			0.453
≥60	32 (57.1)	27 (50.0)	
<60	24 (42.9)	27 (50.0)	
Sex			0.580
male	54 (96.4)	53 (98.1)	
female	2 (3.6)	1 (1.9)	
Smoking status			0.391
Current or former	43 (76.8)	45 (83.3)	
Never	13 (23.2)	9 (16.7)	
Alcohol abuse			0.216
Yes	45 (80.4)	48 (88.9)	
No	11 (19.6)	6 (11.1)	
Stage			0.373
III	7 (12.5)	4 (7.4)	
IV	49 (87.5)	50 (92.6)	
Tumor classification			0.794
2	9 (16.1)	11 (20.4)	
3	18 (32.1)	18 (33.3)	
4	29 (51.8)	25 (46.3)	
Node classification			0.403
0	10 (17.9)	5 (9.3)	
1	4 (7.1)	2 (3.7)	
2	39 (69.6)	45 (83.3)	
3	3 (5.4)	2 (3.7)	
Primary site			0.177
Orbital Fissure	35 (62.5)	42 (77.8)	
The posterior region of the cartilaginous ring.	8 (14.3)	6 (11.1)	
The posterior pharyngeal wall	13 (23.2)	6 (11.1)	
Pathological differentiation			0.952
Highly differentiated	13 (23.2)	12 (22.2)	
moderately differentiated	23 (41.1)	25 (46.3)	
poorly differentiated	19 (33.9)	16 (29.6)	
undifferentiated	1 (1.8)	1 (1.9)	

### Efficacy

#### CR rate, ORR, DCR, downstaging rate and pCR rate

According to the RECIST 1.1 criteria for evaluating solid tumors (**Table 3**), 4 patients in Group A achieved complete remission (CR) (7.1%), 41 (73.2%) patients had a partial response (PR), 9 (16.1%) patients had a stable disease (SD), and 2 (3.6%) patients had a progressive disease (PD). The objective response rate (ORR) was 80.3%, and the disease control rate (DCR) was 96.4% for Group A.

**Table 3:** Response to Group A and Group B (Imaging assessment) [n (%)].

Response of Primary Tumor to Treatment	Treatment Group		P value
	A (n=56)	B (n=54)	
CR	4 (7.1)	1 (1.85)	
PR	41 (73.2)	43 (79.6)	
SD	9 (16.1)	9 (16.7)	
PD	2 (3.6)	1 (1.85)	
ORR	45 (80.3)	44 (81.5)	0.881
DCR	54 (96.4)	53 (98.1)	1.000

CR complete response, PR partial response, SD stable disease, PD progressive disease, ORR objective response rate, DCR disease control rate.

Group B achieved CR in 1 (1.85%) case, PR in 43 (79.6%) cases, SD in 9 (16.7%) cases, and PD in 1 (1.85%) case. The ORR was 81.5%, and the DCR was 98.1% for Group B. **Table 3** shows that the ORR and DCR of the two groups were similar and that there was no significant difference in either ORR or DCR between groups (both P>0.05).

Excluding patients whose imaging assessment was PD, a total of 107 patients underwent pathological assessment after treatment, including 54 patients in Group A and 53 in Group B. From **Table 4** we can see that once again there was no statistically significant difference in MPR (major pathological response) between the two groups (p=0.928), but the rate of pCR in Group A was much higher than that in Group B, at 37.5% vs. 7.4%, p<0.001.

**Table 4:** Response to Group A and Group B (Pathological assessment) [n (%)].

Response of Primary Tumor to Treatment	Treatment Group		P value
	A (n=56)	B (n=54)	
pCR	21 (37.5)	4 (7.4)	<0.001
MPR	46 (82.1)	44 (81.5)	0.928

pCR pathological complete response, MPR major pathological response.

**Figure 1:**
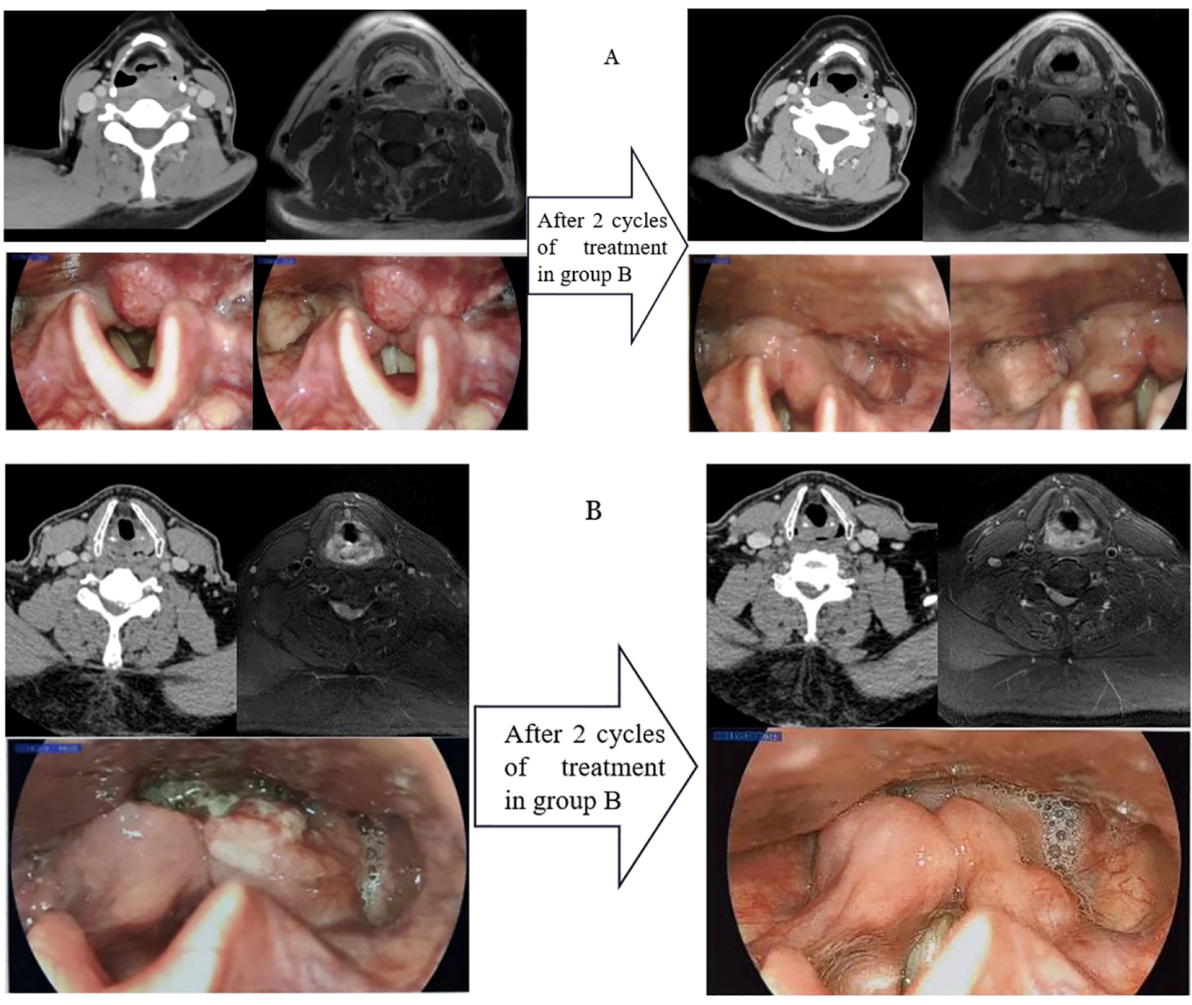
A typical case of immunotherapy combined with chemotherapy as neoadjuvant treatment [Group **(A)** and Group **(B)**], with CT, MRI, and laryngoscope images of the primary tumor before and after two cycles of treatment.

The difference in tumor downstaging between the two groups was analyzed as well, and the typical clinical examples of tumor downstaging after treatment are shown in **Figure 1**. **Table 5** shows that the rate of primary tumor downstaging in Group A patients with locally advanced hypopharyngeal cancer was significantly higher than that of Group B (76.8% vs. 38.9%, p < 0.0001. Taking into account the significant difference in pCR rate between Group A and Group B, the relevant factors in pCR patients and non-pCR patients in Group A were subsequently analyzed for differences. These results (**Table 6**) show that there were no significant differences in age, smoking status, drinking status, clinical stage, TN stage, anatomical site, tumor pathological differentiation degree, or CPS score between pCR and non-pCR patients (all p>0.05).

**Table 5:** The tumor downstaging rate in Group A and Group B after treatment [n (%)].

Tumor downstaging	Treatment Group		P value
	A (n=56)	B (n=54)	
Yes	43 (76.8)	21 (38.9)	<0.001
No	13 (23.2)	33 (61.1)	

**Table 6:** Analysis of clinical characteristics in Group A between pCR and non-pCR patients. [n (%)].

Characteristics	Group A (n=56)		P value
	pCR (n=21)	Non-pCR (n=35)	
Age			0.265
≥60	10 (47.6)	22 (62.9)	
<60	11 (52.4)	13 (37.1)	
Smoking status			0.806
Current or former	17 (81.0)	26 (74.3)	
Never	4 (19.0)	9 (25.7)	
Alcohol abuse			1.000
Yes	17 (81.0)	28 (80.0)	
No	4 (19.0)	7 (20.0)	
Stage			0.917
III	2 (9.5)	5 (14.3)	
IV	19 (90.5)	30 (85.7)	
Tumour classification			0.856
2	4 (19.0)	5 (14.3)	
3	7 (33.3)	11 (31.4)	
4	10 (47.6)	19 (54.3)	
Node classification			0.412
0	2 (9.5)	8 (22.8)	
1	1 (4.8)	3 (8.6)	
2	16 (76.2)	23 (65.7)	
3	2 (9.5)	1 (2.9)	
Primary site			0.996
Orbital Fissure	13 (61.9)	22 (62.9)	
The posterior region of the cartilaginous ring.	3 (14.3)	5 (14.3)	
The posterior pharyngeal wall	5 (23.8)	8 (22.8)	
Pathological differentiation			0.779
Highly differentiated	6 (28.6)	7 (20.0)	
moderately differentiated	8 (38.1)	15 (42.8)	
poorly differentiated	7 (33.3)	12 (34.3)	
undifferentiated	0 (0.0)	1 (2.9)	
CPS			0.207
≥20	11	13	
1≤CPS<20	10	18	
<1	0	4	

#### PFS and OS

**Figure 2:**
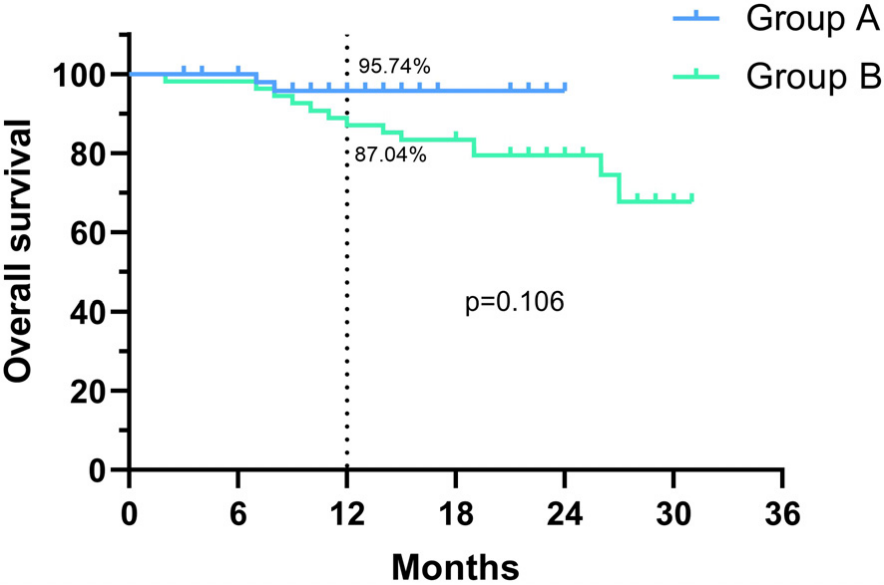
Comparison of overall survival (OS) between the two groups.

**Figure 3:**
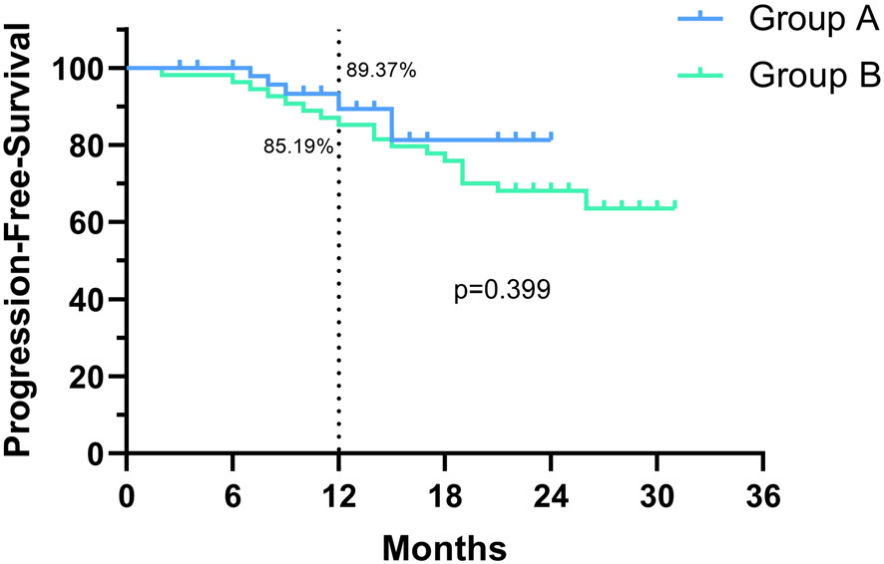
Comparison of progression-free survival (PFS) between the two groups.

The median follow-up time was 10 months in group A and 23 months in group B. Kaplan-Meier analysis and log-rank tests were performed on both groups based on their follow-up data, and no significant differences in OS rate or PFS rate were found between the two groups. Due to the short follow-up time, however, the median OS and median PFS were not reached in both groups. As shown in **Figure 2**, the 1-year OS rate was 95.7% in Group A and 87.0% in Group B (p=0.106, HR=0.340; 95% CI: 0.114-1.013), and as shown in **Figure 3**, the 1-year PFS rate was 89.4% in Group A and 85.2% in Group B (p=0.399, HR=0.675; 95% CI: 0.275-1.659).

**Figure 4:**
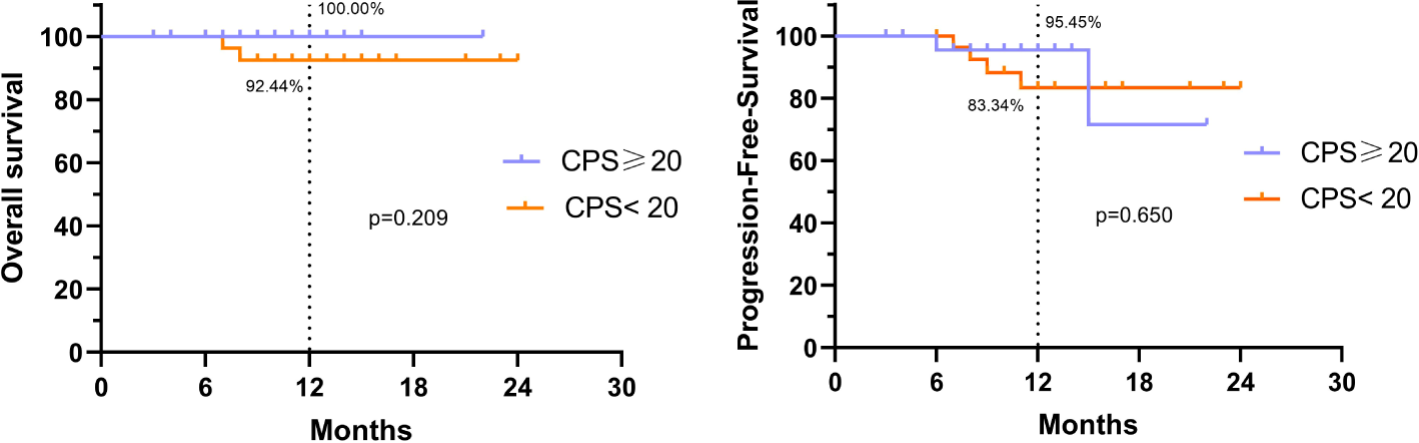
OS rate and PFS rate of patients with different CPS scores in Group A.

Survival analyses were performed for patients in Group A with CPS of ≥20 and those with CPS of <20, and no significant differences in OS rates (P=0.209) or PFS rates (P=0.650) were found between the two groups (**Figure 4**).

#### Laryngeal function preservation

**Table 7** shows that the laryngeal function preservation rate in Group A was 85.7% (48/56), with a majority of patients maintaining satisfactory speech, swallowing, and respiratory functions. In contrast, only 6 out of 54 patients (66.7%) in Group B retained their laryngeal function. Moreover, the disparity in laryngeal function preservation rate between the two groups was statistically significant (p=0.019). Additionally, more patients in Group A received radiotherapy or transoral endoscopic microlaryngeal function-preserving surgery, and more patients in Group B received transcervical partial laryngectomy (p<0.001) (**Table 8**).

**Table 7:** Comparison of Larynx function preservation rate between the two groups after treatment.

Larynx function preservation	Treatment Group [n (%)]		P value
	A (n=56)	B (n=54)	
YES	48 (85.7)	36 (66.7)	0.019
NO	8 (14.3)	18 (33.3)	

**Table 8:** Comparison of subsequent therapeutic approaches in patients with preserved laryngeal function.

Therapeutic approaches	Treatment Group [n (%)]		P value
	A (n=48)	B (n=36)	
Supporting laryngoscope or radiation therapy	37 (77.1)	9 (25)	<0.001
Partial laryngectomy	11 (22.9)	27 (75)	

#### Safety and toxicity

The adverse events related to the drugs were evaluated during the treatment period according to the Common Terminology Criteria for Adverse Events (CTCAE) version 5.0. Among the 56 patients in Group A, 55 (98.2%) experienced adverse events of various degrees, with 46 patients experiencing level1-2 adverse events. There were 46 cases of blood and lymphatic system disorders (mainly anemia, neutropenia, and thrombocytopenia), 52 cases of metabolism and nutrition disorders (mainly hypocalcemia, hyponatremia, and hypokalemia), 4 cases of endocrine disorders (2 cases of hyperglycemia, 2 cases of hyperthyroidism), 6 cases of gastrointestinal disorders, 5 cases of transient fever, 2 cases of skin and subcutaneous tissue disorders, 4 cases of liver function abnormalities, and 1 case of kidney function abnormality (proteinuria). Nine patients had level 3-4 adverse events, including 7 cases of blood and lymphatic system disorders (2 cases of anemia, 4 cases of neutropenia, 1 case of thrombocytopenia), 1 case of metabolism and nutrition disorder (hyponatremia), and 1 case of endocrine disorder (hyperglycemia). Among the 54 patients in Group B, 79.6% (43/54) experienced adverse events, with 8 patients experiencing level 3-4 adverse events. Overall, the incidence of adverse events in Group A was higher than that in Group B (**Table 9**), and the difference was statistically significant (p=0.002). However, when comparing the differences in the grade of adverse events between the two groups (**Table 10**), there was no statistical difference in the incidence of level 3-4 adverse events between the two groups (p=0.771).

**Table 9:** Comparison of the incidence of adverse events between the two groups.

Treatment Group	Number	Number of Adverse Events [n (%)]	P value
A	56	55 (98.2)	0.002
B	54	43 (79.6)	

[n (%)].

**Table 10:** Comparison of the grade of adverse events among patients in the two groups.

Adverse Events	Number of Adverse Events [n (%)]		P value
	A (n=55)	B (n=43)	
Grade1-2	46 (83.6)	35 (81.4)	0.771
Grade3-4	9 (16.4)	8 (18.6)	

[n (%)].

**Figure 5:**
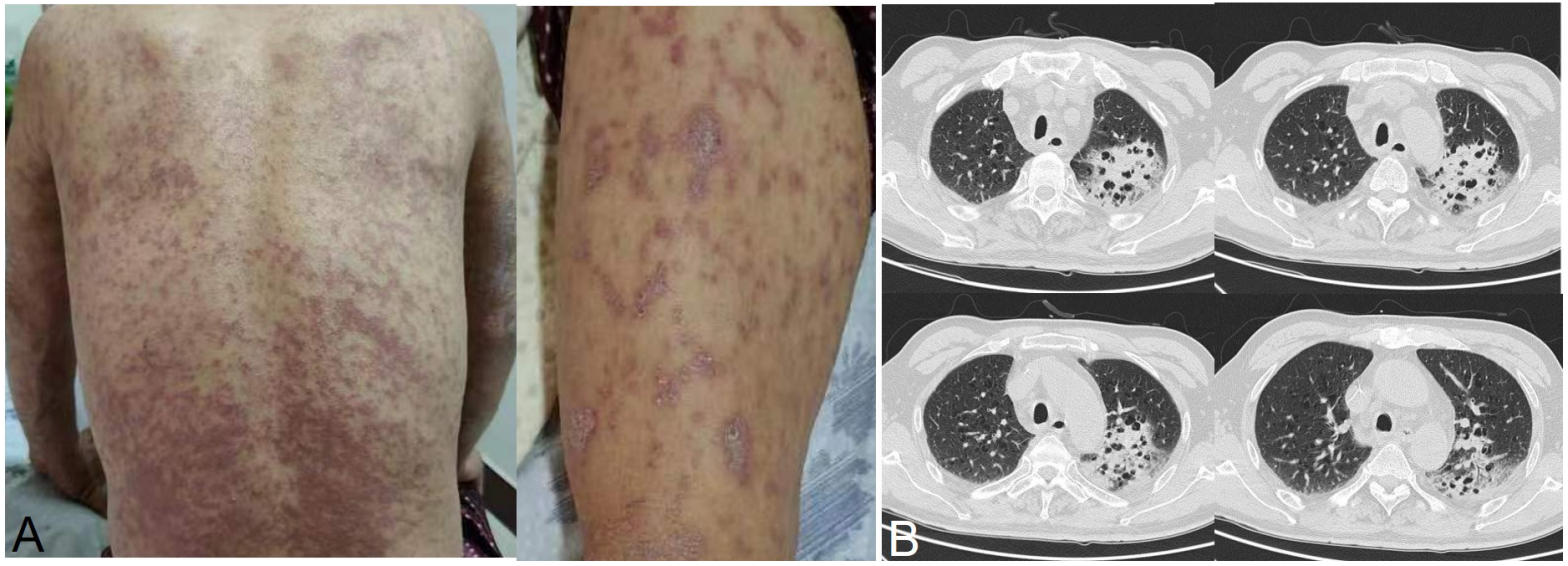
Patients who experienced severe adverse events during immunotherapy combined with chemotherapy. **(A)** Systemic rash; **(B)** immune-mediated pneumonitis.

During the collection of clinical data for locally advanced hypopharyngeal cancer patients at our hospital, we found that there were patients who stopped taking the drugs due to severe adverse reactions in both groups, including those who did not complete the full 2-cycle neoadjuvant treatment. For example, Patient 1, a male patient aged 53, had TNM stage T3N1M0 cancer, a CPS score of 20, and developed systemic eruptions after one cycle of treatment (**Figure 5A**). After stopping the treatment, no new eruptions occurred. Patient 2, a male patient aged 66, had T4aN2cM0 cancer and a CPS score of 2, and developed an immune-mediated pneumonitis after one cycle of treatment (**Figure 5B**). This patient immediately stopped the treatment and received corticosteroid shock therapy instead.

## Discussion

Due to continuous innovation in treatment concepts and technology in recent years, HPSCC has seen an improvement in both outcomes and prognosis under multi-modal treatment strategies that combine surgery, chemotherapy, targeted therapy, and radiotherapy. However, for locally advanced hypopharyngeal squamous cell carcinoma, surgery can lead to the loss of important structures and functions, making preoperative neoadjuvant therapy an indispensable first step. To this end, efforts have already been made to explore better treatment options for locally advanced and recurrent/metastatic hypopharyngeal cancer (LA HPSCC). LA HPSCC is characterized by extensive tumor involvement, local invasion, and regional lymph node metastasis, as well as a high risk of local recurrence and distant metastasis (16, 17). TPF induction chemotherapy has shown better treatment responses and lower toxicity in advanced hypopharyngeal cancer in clinical trials, reduced risk of distant metastasis, improved survival outcomes, and preserved laryngeal function (18). the 5-year follow-up study in the TAX324 clinical trial showed that the total OS of the TPF group was higher than that of the PF group (52% vs. 42%), and the progression-free survival rate (PFS) was better (45% vs. 34%) as well, though this difference was not statistically significant (19).

For R/M HNSCC, in 2008 (20) researchers reported that targeted combined chemotherapy (EXTREME regimen) had better OS and ORR compared to chemotherapy alone (platinum-based chemotherapy plus fluorouracil) and that the EXTREME regimen significantly prolonged the median survival period from 7.4 months 10.1 months (HR=0.80; 95% CI, 0.64-0.99; P=0.04). in addition, cetuximab combined with radiation therapy has also shown long-term survival benefits in locally advanced head and neck squamous cell carcinoma (21). In recent years, studies on neoadjuvant therapy combining targeted treatment with TP have not yielded significant positive results, however (22). In one phase III clinical trial (NCT01434394), among the patients completed all treatment, the difference was not significant between the cetuximab + TP and control arms in terms of OS (HR=0.91, P=0.61), DFS (HR=0.96, P=0.82), or DSS (disease-special survival) (HR=0.92, P=0.69). However, all of the above studies were conducted on oral/oropharyngeal squamous cell carcinoma, and there remains a paucity of data on HPSCC. In this study, the data from Group B suggest that it is not effective in improving pCR and CR compared to existing chemotherapy neoadjuvant treatment data, and therefore its clinical application still requires caution.

Recent clinical trials of PD-1 inhibitors in head and neck cancer have shown good efficacy. In 2014, the KEYNOTE-012 study first confirmed the survival benefit in the PD-L1-positive R/M HNSCC population (23), and in 2016, Ferris R L published the results of the phase III trial Checkmate-141, showing that the ORR of nivolumab in R/M HNSCC patients was 13.3%, with was a significant OS benefit in HNSCC patients whose platinum-based therapy had failed (24). The KEYNOTE-040 study, similarly to CheckMate-141, showed that the median OS of the pembrolizumab group was longer than that of the standard treatment group (8.7 months vs. 7.1 months), especially in the subgroup with tumor positive score ≥ 50% (11.6 months vs. 6.6 months) (25). Additionally, the KEYNOTE-048 study of first-line treatment for R/M HNSCC that included 882 R/M HNSCC patients, pembrolizumab monotherapy was found to extend OS in R/M HNSCC patients with combination positive score ≥ 1 (median survival time 12.3 months vs. 10.3 months), and pembrolizumab plus platinum and fluorouracil improved the OS of the total study population significantly compared to the standard EXTREME regimen (median OS 13.0 months vs. 10.7 months) (7).

The above research findings suggest that PD-1 monotherapy and combination chemotherapy are both safe and effective for treating R/M HNSCC and that they can significantly improve OS compared to the EXTREME regimen. However, the phase III clinical trial data (26) for the neoadjuvant treatment of LA HNSCC show that compared to concurrent chemoradiotherapy, there is no improvement in EFS or OS. Whether immune neoadjuvant therapy can improve patients’ long-term survival still needs further investigation. Previous phase II clinical trials (11, 13, 15) have shown that the combination of PD-1 and TP regimen (2-3 cycles) has an ORR of over 75% and a pCR rate of 36.4-50% in LA HNSCC, with postoperative LPR of 80% or more. This may be useful in determining the best treatment strategy for LA HPSCC.

There have been many reports of comprehensive treatments for head and neck squamous cell carcinoma, including laryngeal cancer, hypopharyngeal cancer, oral cancer, and oropharyngeal cancer. Our center takes advantage of its clinical cases in order to analyze and compares the efficacy differences between immunotherapy combined with chemotherapy as a neoadjuvant treatment and targeted therapy combined with chemotherapy as a neoadjuvant treatment for locally advanced hypopharyngeal cancer specifically. According to the information we have reviewed, there has yet to be a study on the efficacy difference analysis of these two treatment schemes for monospecific locally advanced hypopharyngeal cancer at the international level.

In an effort to remedy this gap in the research, we conducted a study to compare PD-1 inhibitors (Group A) combined with chemotherapy to targeted therapy combined with chemotherapy (Group B) and found that the ORR and DCR were similar between the two groups, at 80.3% vs. 81.5% and 96.4% vs. 98.1%, respectively. However, the immunotherapy plus chemotherapy group had a significantly higher pCR rate (37.5% vs. 7.4%). As a result, the primary tumor downstaging rate in Group A was significantly higher than that in Group B (76.8% vs. 38.9%). Furthermore, 1-y OS rate in Group A was 95.7% compared to 87.0% in Group B, and the 1-y PFS rate was 89.47% vs. 85.2%. The laryngeal function preservation rate in Group A was higher as well, at 85.7% compared to 66.7% for Group B (p=0.019). These findings are consistent with those reported in a recent phase II prospective clinical trial published on NC (27), where the ORR was 82.4% (42/51), and the 2-year OS and PFS rates were 83.0% and 77.1%, respectively.

In previous studies, PD-1 inhibitors have been shown to be safe with good tolerability (28), and in the present study, although the incidence of adverse reactions was higher in Group A, most were level 1-2, and there was no difference in the incidence of level 3-4 adverse reactions between the two groups (p=0.771). This indicates that PD-1 inhibitors do not exacerbate severe adverse events associated with chemotherapy. Furthermore, although immunotherapy is safe for most patients, it can cause severe adverse reactions such as immune-mediated pneumonitis, acute kidney injury, immune-mediated myocarditis, and even hyperprogressive disease (HDP) in a small number of patients (29, 30).

For all of the encouraging results discussed above, however, immune-combined chemotherapy neoadjuvant treatment is still in the exploration stage, with many questions yet to be answered. The first is whether immune-combined chemotherapy neoadjuvant treatment can improve the long-term benefits of patients with hypopharyngeal cancer, and to answer this more clinical trial results, and more long-term follow-up analysis are needed. Second, the predictive biomarkers for the efficacy of neoadjuvant immunotherapy are not clear, and the commonly used CPS (combined positive score), TPS (tumor cell Proportion Score), and TMB (tumor mutation burden) (31, 32), as well as other detection indicators, have limited predictive ability for efficacy. For example, in this study, the patients with SD/PD treatment effect assessments accounted for about 27.3% (3/11) of those with CPS≥20, and the results (**Table 6**) showed that there was no significant difference in CPS score between pCR patients and non-pCR patients (p>0.05). Therefore, in the future, we need to analyze various indicators more comprehensively and further explore more accurate efficacy prediction indicators. In addition, the question of how to identify immune hyperprogress and pseudoprogression, avoid immune inflammatory storm, and reduce more serious adverse reactions still needs answered.

Due to the single-center design of this study, the number of cases included was limited, and the follow-up time was relatively short, which may have resulted in certain biases in the survival analysis. Additionally, the study was retrospective in design, which inherently introduces the problems of selection bias and missing data, and our results thus need to be confirmed by prospective clinical trials and case-control studies. Through a retrospective case summary, this study analyzed and compared the differences in immunotherapy combined with chemotherapy for neoadjuvant treatment and targeted therapy combined with chemotherapy for local advanced hypopharyngeal cancer. Compared with targeted therapy combined with chemotherapy for neoadjuvant treatment, immunotherapy combined with chemotherapy for neoadjuvant treatment can prolong the PFS and OS of patients, and the combined treatment does not increase the incidence of 3-4 grade adverse reactions, ensuring safety that is controllable. This study provides reference for the exploration and application of neoadjuvant immunotherapy in hypopharyngeal cancer.

## Conclusion

Compared to the combination of targeted therapy and chemotherapy for neoadjuvant treatment, the combination of immunotherapy and chemotherapy for neoadjuvant treatment in the treatment of locally advanced hypopharyngeal cancer showed better efficacy in increasing the pCR rate of patients, prolonging their overall survival time and progression-free survival time, and dose not increase the incidence of level 3-4 adverse reactions, with controllable safety. Based on these results we have already begun conducting a phase III neoadjuvant trial for PD-1 inhibitors in locally advanced head and neck squamous cell carcinoma in an effort to validate the safety and efficacy of immunotherapy reported here (NCT06102395).

## Data Availability

The raw data supporting the conclusions of this article will be made available by the authors, without undue reservation.
